# Lipid A-based affinity biosensor for screening anti-sepsis components from herbs

**DOI:** 10.1042/BSR20130103

**Published:** 2014-05-23

**Authors:** Jie Yao, Yiguo Chen, Ning Wang, Dongneng Jiang, Jiang Zheng

**Affiliations:** *Medical Research Center, Southwest Hospital, Third Military Medical University, Chongqing 400038, People's Republic of China

**Keywords:** affinity biosensor, Chinese herbs, lipid A, sepsis, CFU, colony-forming units, DMEM, Dulbecco’s modified Eagle’s medium, KM, KunMing, LAL, *Limulus* amoebocyte lysate, LB, Luria–Bertani, LPS, lipopolysaccharide, NS, normal saline, OG, *n*-octyl β-D-glucopyranoside, PMB, polymyxin B, PSA, *Paeonia suffruticosa* Andr, SIRS, systemic inflammatory response syndrome, SPF, specific pathogen free, TNFα, tumour necrosis factor α

## Abstract

LPS (lipopolysaccharide), an outer membrane component of Gram-negative bacteria, plays an important role in the pathogenesis of sepsis and lipid A is known to be essential for its toxicity. Therefore it could be an effective measure to prevent sepsis by neutralizing or destroying LPS. Numerous studies have indicated that many traditional Chinese medicines are natural antagonists of LPS *in vitro* and *in vivo*. The goal of this study is to develop a rapid method to screen anti-sepsis components from Chinese herbs by use of a direct lipid A-based affinity biosensor technology based on a resonant mirror. The detergent OG (*n*-octyl β-D-glucopyranoside) was immobilized on a planar non-derivatized cuvette which provided an alternative surface to bind the terminal hydrophilic group of lipid A. A total of 78 herbs were screened based on the affinity biosensor with a target of lipid A. The aqueous extract of PSA (*Paeonia suffruticosa* Andr) was found to possess the highest capability of binding lipid A. Therefore an aqueous extraction from this plant was investigated further by our affinity biosensor, polyamide chromatography and IEC–HPLC. Finally, we obtained a component (PSA-I-3) from *Paeonia suffruticosa* Andr that was evaluated with the affinity biosensor. We also studied the biological activities of PSA-I-3 against sepsis *in vitro* and *in vivo* to further confirm the component we screened with the biosensor. *In vitro*, we found that PSA-I-3 could decrease TNFα (tumour necrosis factor α) release from RAW264.7 cells induced by LPS in a dose-dependent manner. *In vivo*, it increased remarkably the survival of KM (KunMing) mice by challenging both lethal-dose LPS and heat-killed *Escherichia coli* compared with control groups. Our results suggest that the constructed affinity biosensor can successfully screen the anti-sepsis component from Chinese herbs.

## INTRODUCTION

Sepsis is a complex syndrome that is defined as the presence of infection plus SIRS (systemic inflammatory response syndrome) [1]. In the USA, sepsis syndrome is responsible for a tenth of all deaths. The mortality rate for inpatients with sepsis is approximately 30–55% and higher for the elderly [2,3]. Moreover, the conditions of sepsis are worse in developing countries owing to poor healthcare and environment. Although Gram-negative bacteria are no longer the number one cause of sepsis in recent years, as people now pay more attention [4,5], infections still result in severe outcomes such as sepsis, severe sepsis and septic shock in most of these critically ill populations who do not receive appropriate and timely therapy [6].

LPS (lipopolysaccharide) is an outer membrane component of Gram-negative bacteria and more than 2×10^6^ LPS molecules comprise a polysaccharide part, O-antigen and core regions, and a lipid anchor, called lipid A, known to be essential for its toxicity [7]. Numerous studies have indicated that LPS triggers pathogenesis of sepsis by recognizing TLR4 (Toll-like receptor 4) and CD14 on the surface of cells; so an effective way to prevent sepsis could be by neutralizating LPS [8–12]. In recent years, many therapeutiic strategies were taken against LPS including McAbs, polyclonal antibodies, leucocyte proteins (BPI), PMB (polymyxin B), mimic peptides, etc. [13–19]. Unfortunately, to date, there is not yet a safe and effective agent to protect victims from sepsis caused by LPS.

In China, Chinese herbs have been used as drugs for critically ill patients for thousands of years. There is evidence that also suggests that many Chinese traditional herbs possess an anti-sepsis function [20]; however, traditional herbs are so complex that their clinical applications are restricted greatly. We have thus become interested in exploring a method with advantages of being sensitive, fast, accurate, label-free, simple and quantitative. In the present study, lipid A was immobilized on the OG (*n*-octyl β-D-glucopyranoside)-coated surface of a non-derivatized cuvette to construct an affinity biosensor developed to screen anti-sepsis herb technology in 2004. This identified some anti-sepsis fractions or monomers from *Radix Paeoniae* Rubra, *Scutellaria baicalensis* Georgi and *Terminaliachebula* Retz in the following years [21–23]. Herein, we found that 12 aqueous extractions from 78 Chinese herbs could bind greatly to lipid A which was immobilized on to a non-derivatized cuvette of the affinity biosensor and the non-tannin aqueous extraction from PSA (*Paeonia suffruticosa* Andr) possessed the highest lipid A-binding ability after consuming the indicated LPS concentration. Therefore an aqueous extraction from this plant was investigated further by an affinity biosensor, polyamide chromatography and IEC–HPLC. Finally, we obtained a fraction (PSA-I-3) from *Paeonia suffruticosa* Andr and investigated its anti-sepsis activities *in vitro* and *in vivo*.

## MATERIALS AND METHODS

### Reagents

LPS from *Escherichia coli* O111:B4 and lipid A from *Salmonella* Re 595 were obtained from Sigma Chemicals. Mouse TNFα (tumour necrosis factor α) ELISA kits were obtained from Biosource International. The kinetic turbidimetric LAL (*Limulus* amoebocyte lysate) kit was obtained from Zhanjiang A & C Biological Ltd., and gelatine was purchased from Sino-pharm Chemical Reagent Co., LtdS.

### Chinese herbs

Twelve Chinese herbs were identified in the Chongqing Academy of the Chinese Materia Medica (Chongqing, China) which were purchased from Sichuan Province.

### Mice

Eighty KM (KunMing) mice (4–6 weeks old), with an equal number of males and females at random, were obtained from the Experimental Animal Center of Chongqing Medical University (Chongqing, China). The weight of the mice was 20±2 g. The mice were housed in SPF (specific pathogen-free) conditions until use, and in agreement with principles stated in the Guide for the Care and Use of Laboratory Animals. National Research Council Publication, 1996 edition.

### Cell line and culture

The mouse macrophage cell line RAW264.7 was obtained from American Type Culture Collection (Manassas, VA), and was grown in DMEM (Dulbecco's modified Eagle's medium) supplemented with 10% (v/v) FBS (HyClone), 2 μM glutamine, 100 units/ml penicillin and 100 μg/ml streptomycin at 37°C in a humidified 5% (v/v) CO_2_ incubator. In each experiment, 10^6^ cells/well were used.

### Preparation of bacterial strains

*E. coli* (ATCC 25922) maintained in our laboratory were subcultured on the LB (Luria–Bertani) agar plate for 18 h, and then passaged into LB broth (containing 10 g tryptone, 10 g NaCl and 5 g yeast extract per litre) and cultivated aerobically in a 50 ml volume at 37°C in a heated, shaking environmental chamber for 18 h and transferred to 1000 ml of fresh LB broth for another 18 h. They were harvested by centrifugation. Finally, the bacteria were resuspended in sterile NS (normal saline) and killed at 100°C in hot water for 30 min.

### Preparation of aqueous extracts of 12 Chinese herbs

Aqueous extracts of 12 Chinese herbs were prepared as described in [22]. Briefly, 2 g of 12 herbs were added to 10 ml distilled water after pulverization and heated in a water bath at 100°C for 1 h. The 12 aqueous extractions were centrifuged at 4°C at 5000 rev/min for 3 min and each supernatant was collected after filtration for the next assays.

### Removing tannins from 12 Chinese herbs

The 12 supernatants as stated above were co-incubated with excessive gelatine (4%, w/v) at 50°C for 1 h. The supernatants, after centrifugation at 4°C, 10 000 rev/min for 5 min, were collected and stored at 4°C for the following assays.

### Consumption assays of the indicated LPS concentration

Approximately 50 μl supernatants, after removing tannins, were co-incubated in the presence or absence of 50 μl LPS (20, 50 ng/ml) at 30°C for 30 min. After centrifugation at 4°C, 5000 rev/min for 3 min the supernatants were collected and stored at 4°C for use in lipid A-binding assays.

Lipid A was immobilized on to the surface of a non-derivatized cuvette according to the manufacturer's instruction (Thermo Labsystem) as described in [22]. Briefly, 5 ml of 12 supernatants after consumption of the indicated LPS concentration was assayed for its binding activity to lipid A as described in [22]. Data are shown in Table 1.

**Table 1:** Lipid A-binding activity of aqueous extracts bound to the indicated LPS concentration after removing tannins

	Response unit (arc-sec)		
Extract	50 ml (NS)	20 ng (LPS)	50 ng (LPS)
*Scutellaria baicalensis* Georgi	190	133	129
*Terminaliachebula Retz*	473	255	211
*Fructus crataegi*	580	348	227
*Biota orientalis*	638	573	215
*Rhizoma coptidis*	419	278	219
*Radix et Rhizoma Rhei*	810	589	564
*Chinese White Olive*	581	228	135
*Tripterygium Wilfordii*	427	368	298
*Radix Paeoniae Rubra*	511	469	244
*Radix Sanguisorbae*	626	555	369
*Caulis Sargentodoxae*	633	583	553
*Paeonia suffruticosa* Andr	952	702	654

### Isolation of lipid A-binding fractions from PSA

**Figure 1:**
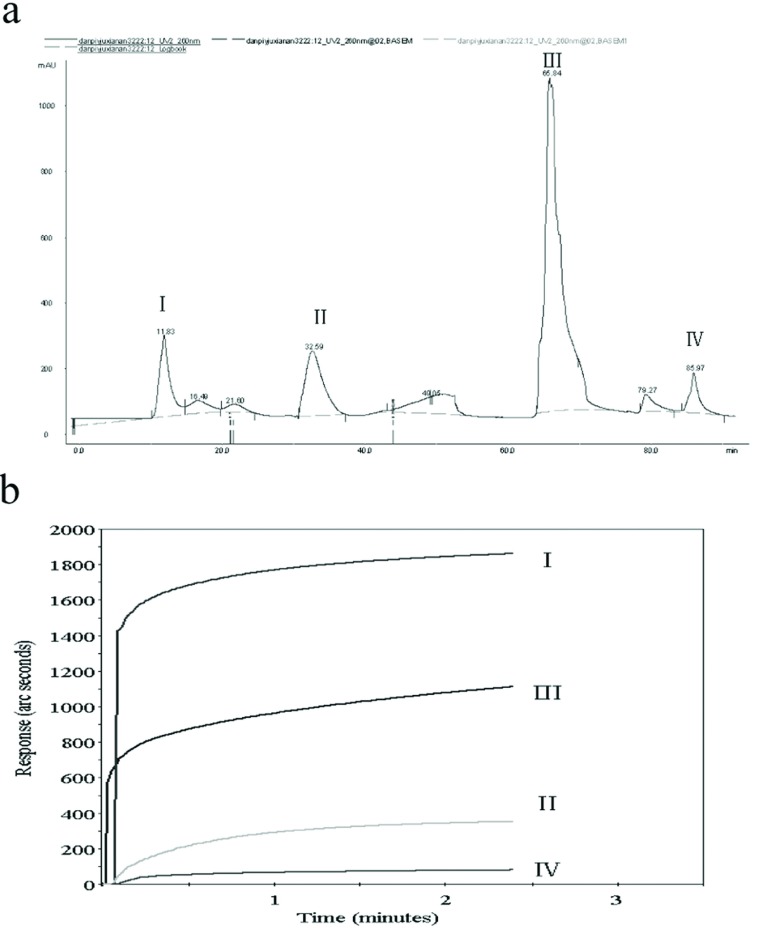
Aqueous extracts from PSA and their lipid A-binding abilities (**a**) Isolation with polyamide chromatography. Aqueous extract of PSA was passed over column chromatography with polyamide (80–100 mesh, 45 mm×250 mm), and eluted with distilled water, absolute ethanol and NH_3_.H_2_O (1%), respectively. We obtained together four fractions, named PSAI–IV. (**b**) Five microlitres of PSAI–IV (10 μg/ml) were assayed for binding activity to lipid A. Lines I–IV represented their lipid A-binding abilities.

Approximately 1 kg PSA was boiled in distilled water at 80°C for 1 h via regurgitant extraction and tannins were removed by excessive gelatine (4%) at 50°C for 1 h before filtration. The filtrate was centrifuged at 4000 ***g*** for 30 min and the supernatant was collected, which was passed over column chromatography with polyamide (80–100 mesh, 45×250 mm), eluted with distilled water, absolute ethanol and NH_3_.H_2_O (1%). The elution was collected and concentrated by rotary evaporation (BUCHI Rotavapor R205) to collectively obtain four fractions, named PSAI–IV. Briefly 5 μl PSAI–IV (10 μg/ml) was assayed for its binding activity to lipid A, as described in [22]. The results indicated that the first fraction (PSA-I) bound more markedly to lipid A than the others, and at more than 1800 arc-sec (Figure 1b).

**Figure 2:**
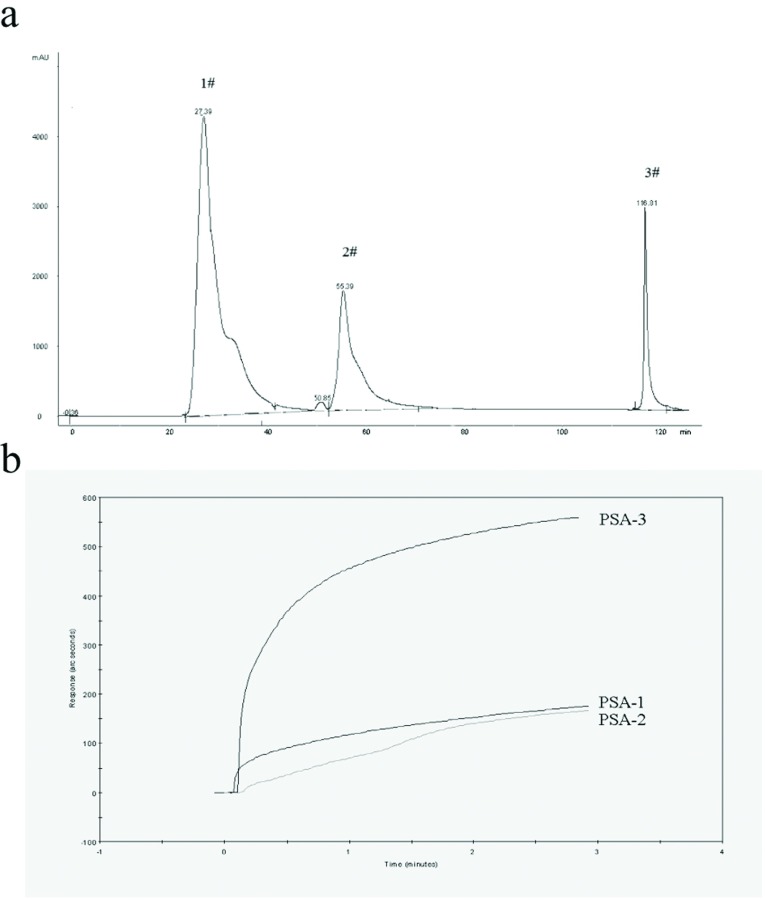
The elution from PSA-I via IEC–HPLC and their lipid A-binding abilities (**a**) In order to investigate further the anti-LPS characteristics of PSA-I, we purified further the first fraction (PSA-I) via IEC–HPLC. To this end, the fraction (PSA-I) was injected on to an IEC–HPLC system (SPFF 90 μm, GE Helathcare), eluted with acetic acid (pH 4.0), double-distilled water and NH_3_.H_2_O (1%) adding ethanol (1%), respectively. We obtained, in total, three HPLC fractions, named PSA1–3. (**b**) Five microlitres of PSA1–3 (10 μg/ml) were assayed for binding activity to lipid A. Lines 1–3 represented their lipid A-binding abilities.

In order to investigate the anti-LPS characteristics of PSA-I, we further purified the first fraction (PSA-I) via IEC–HPLC. To this end, the fraction (PSA-I) was injected on to the IEC–HPLC system (SPFF 90 μm, GE Healthcare), eluted with acetic acid (pH 4.0), double-distilled water and NH_3_.H_2_O (1%) adding ethanol (1%, v/v), respectively, to obtain three HPLC fractions (PSA1–3). Briefly 5 μl of PSA1–3 (10 μg/ml) was assayed for its binding activity to lipid A as described previously [22]. We found that the third fraction (PSA-I-3) bound markedly to lipid A at up to 550 arc-sec (Figure 2b).

### LPS neutralization by PSA-I-3 *in vitro*

To determine whether PSA-I-3 could antagonize the bioactivity of LPS, we verified LPS neutralization by PSA-I-3 *in vitro* using the *Limulus* test, which is widely used to detect the concentration of microamounts of endotoxin. For this experiment, two concentrations of the PSA-I-3 (1 and 10 μg/ml) were co-incubated in the presence or absence of LPS (10 ng) at 37°C for 30 min; the control experiments were as follows: positive control (1 μg PMB+10 ng LPS), negative control (10 ng LPS) and internal controls (sterile NS). Then 100 μl of each mixture was added to LAL reagent of an equal volume, and the kinetic turbidity was measured using an EDS-99 Tube Reader (Zhanjiang A&C Biological Ltd.).

### Inhibition of TNFα release by RAW264.7 cells induced by LPS

For this experiment, 1×10^5^ RAW264.7 cells (200 μl) were plated on to a 96-well microplate and cultured at 37°C in a humified 5% CO_2_ incubator for 4 h to subconfluency. The cells were pretreated with PSA-I-3 at the indicated dose (45, 90 and 180 μg/ml) for 30 min, followed by replacement with 0.5 ml serum-free DMEM. Cells were then stimulated with and without LPS (100 μg/l) for 4 h. Positive control (10 μg PMB+100 μg/l LPS), negative controls and blank control groups were 100 μg/l LPS and an equal volume of serum-free DMEM, respectively. Supernatants were collected to test the TNFα level using mouse ELISA kits.

### Protection of PSA-I-3 on mice challenged by LPS

A total of 40 mice (KM) were randomly divided into four groups (10 mice per group), mice (KM) were challenged with LPS (18 mg/kg) via the tail vein. Subsequently, PMB (2 mg/kg) and PSA-I-3 (100 mg/kg) were injected as the positive control and treatment groups using identical procedures as stated above. PSA-I-3 (100 mg/kg) alone was used as a blank control. The mice survival was assessed at 6, 12, 24, 36, 48, 60, 72, 96 and 108 h.

### Protection of PSA-I-3 on mice challenged by heat-killed *E. coli*

A total of 40 mice (KM) were randomly divided into four groups (10 mice per group) and were injected via the tail vein as follows: heat-inactivated *E. coli* [6.0×10^9^ CFU (colony-forming units)/kg] alone as the negative control, heat-inactivated *E. coli* (6.0×10^9^ CFU/kg) adding dexamethasome (5 mg/kg) as the positive control, and heat-inactivated *E. coli* (6.0×10^9^ CFU/kg) and immediate subsequent injection of PSA-I-3 (100 mg/kg) as the treatment group. PSA-I-3 (100 mg/kg) alone was the blank control. The general conditions and mice mortalities were assessed at 6, 12, 24, 36, 48, 60, 72, 96 and 108 h.

### Statistical analysis

Statistical comparisons of the survival between the experimental and control groups were made using the χ^2^ test, and others were conducted via independent samples using Student's *t* test with the SPSS 11.0 software package. *P*<0.05 (double-sided) was considered as significant, and *P*<0.01 was considered as very significant.

## RESULTS

### Aqueous extracts of 12 Chinese herbs (after removal of tannins) bound to the indicated LPS concentration

In previous experiments, we found that 12 out of the 78 herbs possess high lipid A-binding activities (more than 200 arc-sec) and the tannins have a great impact on binding between lipid A and Chinese herbs. Therefore we carried out an experiment to remove tannins from Chinese herbs, and then the 12 supernatants, after removal of tannins, were co-incubated in the presence or absence of the indicated LPS concentration. The lipid A-binding assays showed that Radix et Rhizoma Rhei, Caulis Sargentodoxae and PSA still possessed higher response units than that of others, and at more than 500 arc-sec. Furthermore, PSA showed the highest lipid A-binding ability among the 12 herbs (Table 1). So, we further investigated the anti-LPS ability of PSA using an affinity biosensor, polyamide chromatography and IEC–HPLC.

### Lipid A-binding fractions from PSA

To determine which components of PSA could inhibit LPS, we isolated the aqueous extracts and tested their lipid A-binding abilities via polyamide chromatography and an affinity biosensor. As a result, four fractions were obtained from the aqueous extract of PSA (Figure 1a) and bound to lipid A which was immobilized on the surface of a non-derivatized cuvette. We found that the first fraction possessed the higher lipid A-binding activity compared with the other fractions, at more than 1800 RU (arc-sec) (Figure 1b).

To further study the bioactivities of the PSA-I fraction, PSA-I was passed on an IEC–HPLC system (SPFF 90 μm). We collected three HPLC fractions (PSA1–3) (Figure 2a) and measured the lipid A-binding activity. Our results showed that the third fraction (PSA-I-3) bound more significantly to lipid A than other fractions, and at up to 550 arc-sec (Figure 2b). These results suggested that PSA-I-3 perhaps could be an inhibitor of LPS *in vitro* or *in vivo*.

### LPS neutralization and inhibition of TNFα release induced by LPS *in vitro* in RAW264.7 cells

**Figure 3:**
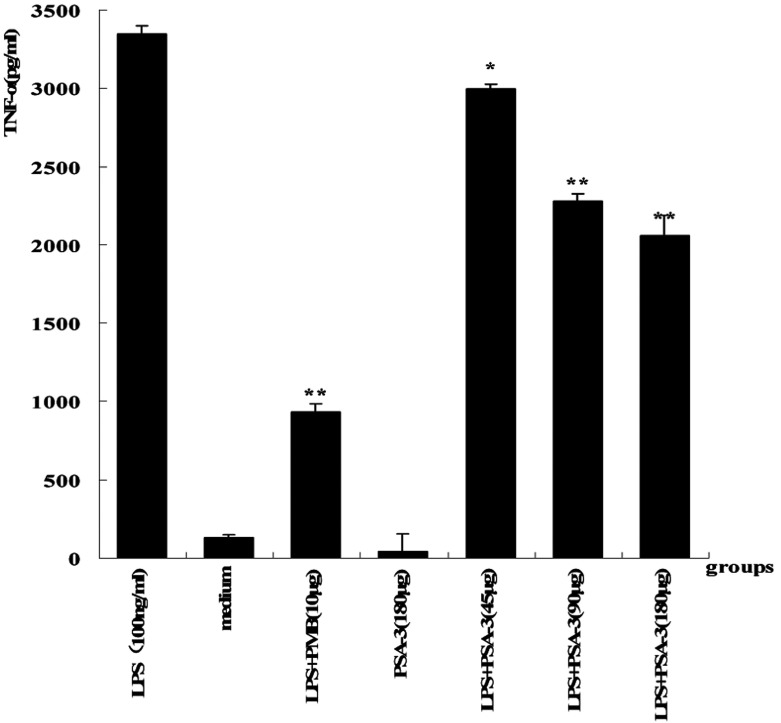
Inhibition of TNFα release from LPS-stimulated RAW264.7 cells For this experiment, 1×10^5^ RAW264.7 cells (200 μl) were plated on to a 96-well microplate and cultured at 37°C in a humified 5% CO_2_ incubator for 4 h to subconfluency. The cells were pretreated with PSA-I-3 at the indicated dose (45, 90 and 180 μg/ml) for 30 min followed by replacement with 0.5 ml serum-free DMEM, and then stimulated with and without LPS (100 μg/l) for 4 h. RAW264.7 cells were also treated with a positive control (10 μg PMB+100 μg/l LPS), and negative and blank control groups were 100 μg/l LPS and an equal volume of serum-free DMEM respectively. Supernatants were collected to test the TNFα level using mouse ELISA kits. The value of TNFα was expressed as mean(pg/ml)±S.D. A *P*-value <0.05 (double-sided) was considered as significant, and a *P*-value <0.01 was considered as very significant. The test was carried out no less than three times.

**Figure 4:**
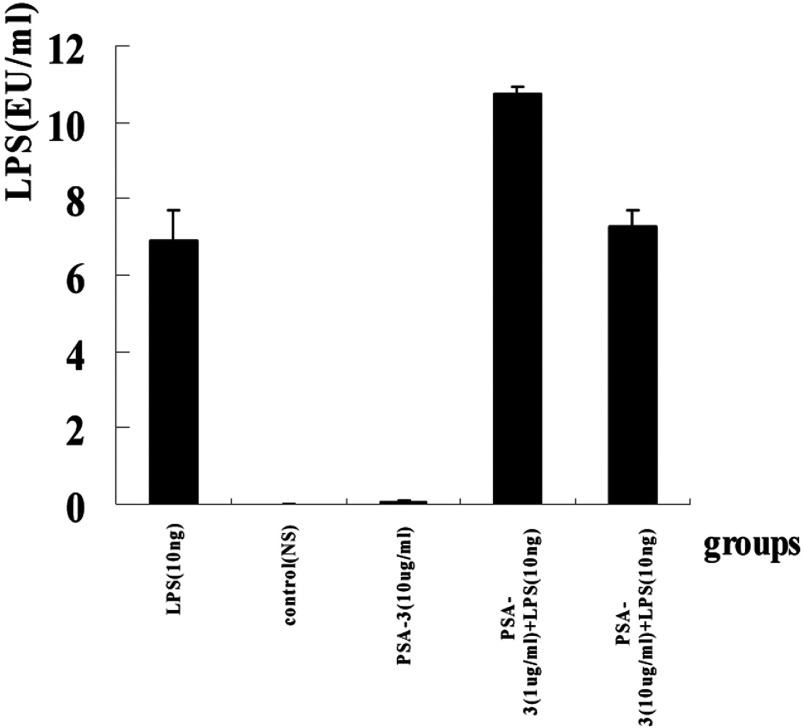
LPS neutralization by PSA-I-3 *in vitro* To determine whether PSA-I-3 could antagonize the bioactivity of LPS, we verified LPS neutralization of PSA-I-3 *in vitro* via a *Limulus* test. To this end, two concentrations of the PSA-I-3 (1 and 10 μg/ml) were co-incubated in the presence or absence of LPS (10 ng) at 37°C for 30 min. Control experiments were as follows: positive control (1 μg PMB+10 ng LPS); negative control, 10 ng LPS; and the internal control was sterile NS. Then 100 μl of each mixture was added to LAL reagent of an equal volume, The kinetic turbidity was measured using an EDS-99 Tube Reader. The value of LPS was expressed as mean (EU/ml)±S.D. A *P*-value <0.05 (double-sided) was considered significant, and a *P*-value <0.01 was considered as very significant. The test was carried out more than three times.

To further evaluate the biological activities of PSA-I-3 against LPS *in vitro*, we measured its abilities to neutralize LPS and to inhibit TNFα release (by LPS challenge) in RAW264.7 cells. In agreement with our hypotheses, the results showed that PSA-I-3 significantly inhibited the LPS-induced RAW264.7 cells to release TNFα in a concentration-dependent manner compared with the negative control (10 ng LPS) (*P*<0.001) (Figure 3). Beyond our expectation, however, PSA-I-3 could not inhibit the lysis of *Limulus* amoebocytes by LPS, it enhanced LAL instead (Figure 4), and the underlying mechanisms remain unclear.

### Protection of PSA-I-3 on mice challenged by LPS and heat-killed *E. coli*

**Figure 5:**
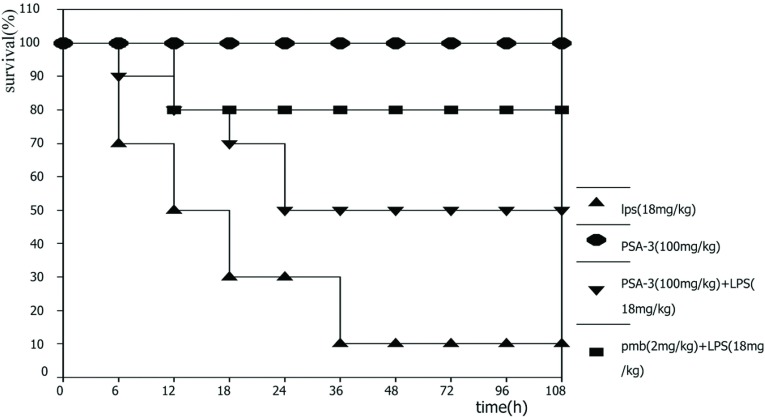
Protection of PSA-I-3 on mice challenged by LPS Forty mice (KM) were randomly divided into four groups (10 mice per group), mice (KM) were challenged with LPS (18 mg/kg) via the tail vein. Subsequently, PMB (2 mg/kg) and PSA-I-3 (100 mg/kg) were injected as the positive control and treatment groups by identical manners as stated above. PSA-I-3 (100 mg/kg) alone was the blank control. The survival of mice was assessed at 6, 12, 24, 36, 48, 60, 72, 96 and 108 h. Statistical comparisons of the survival between the experimental and control groups were made using the χ^2^ test, and a *P*-value <0.01 was considered as very significant. The test was carried out no less than three times.

To further investigate the biological activities of PSA-I-3 against LPS *in vivo*, 40 mice (KM) were injected with PSA-I-3 (100 mg/kg) prior to a lethal challenge with LPS [18 mg/kg, i.v. (intravenously)]. The survival of mice was assessed at 6, 12, 24, 36, 48, 60, 72, 96 and 108 h, respectively. As a result, mice began to die 6 h after challenge with LPS, and the survival of mice with PMB and PSA-I-3 treatment were 80 and 50% after 6 days, respectively. However, nine out of ten mice were dead by challenging with LPS alone approximately 1 week later. Our results suggest that PSA-I-3 protects mice significantly from challenge by a lethal-dose of LPS (*P*<0.005) (Figure 5).

**Figure 6:**
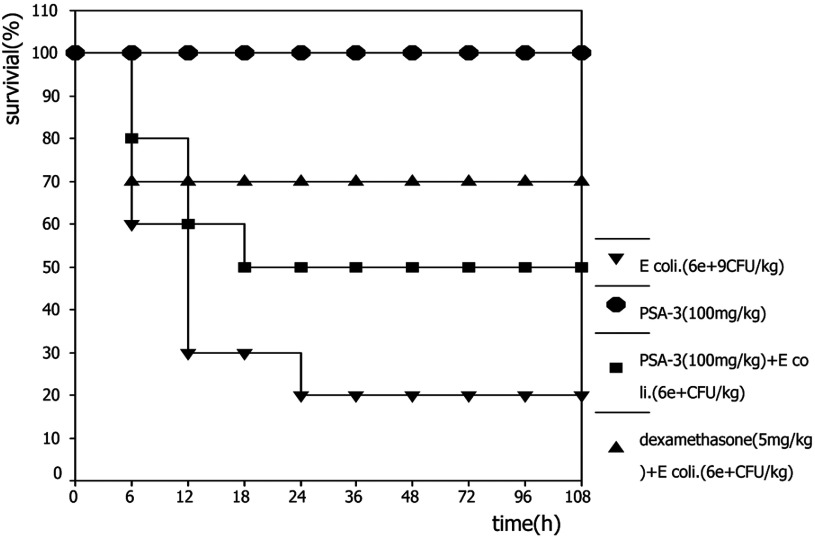
Protection of PSA-I-3 on mice challenged by heat-killed *E coli* Forty mice (KM) were randomly divided into four groups (10 mice per group) and were injected via the tail vein as follows: heat-inactivated *E. coli* (6.0×10^9^ CFU/kg) alone as a negative control, and heat-inactivated *E. coli* (6.0×10^9^ CFU/kg) adding dexamethasone (5 mg/kg) as the positive control. Heat-inactivated *E. coli* (6.0×10^9^ CFU/kg) and immediate subsequent injection of PSA-I-3 (100 mg/kg) was the treatment group. PSA-I-3 (100 mg/kg) alone was a blank control. The general conditions and mice mortalities were assessed at 6, 12, 24, 36, 48, 60, 72, 96 and 108 h. Statistical comparisons of the survival between the experimental and control groups were made using the χ^2^ test, and a *P*-value <0.01 was considered as very significant. The test was carried out no less than three times.

To determine whether PSA-I-3 also protected mice from challenge by natural LPS from *E. coli*, 100 mg/kg PSA-I-3 was administered followed by challenge with heat-killed *E. coli* (6.0×10^9^ CFU/kg) via the tail vein. Post-challenge, the number of dead mice recorded was highest within 12 h and more than 60% of mice were killed in the non-treatment group, and none of the mice that survived died after 24 h in all of the groups. Compared with the non-treatment group, PSA-I-3 improved the survival of experimental animals but this did not show a significant difference (Figure 6).

## DISCUSSION

Previously, we have reported the successful establishment of a platform to screen the active ingredients targeting CpG DNA from traditional Chinese herbs, and found an active component to bind with the platform. In the present study, we also used affinity biosensor technology to establish the platform to screen active ingredients from traditional Chinese herbs targeting lipid-A. It is a different target of LPS, and it was also confirmed that affinity biosensor technology is a rapid and effective method to screen the active fractions from traditional Chinese herbs.

The present study suggested that a lipid A-binding activity peptide possesses endotoxin-neutralizing molecules that could have important clinical applications, which means that a high-binding affinity substance could neutralize endotoxin. However, we have to be sure that the substance is not toxic because it will serve as a treatment for sepsis. For example, PMB possesses high-affinity endotoxin binding, however, it also has severe side effects in humans [24]. Therefore we need to find a new compound with a high affinity to neutralize LPS, but with no toxicity.

PSA (Mudan Pi), belonging to the Paeoniaceae family, is an important traditional Chinese medicine used in many traditional prescriptions. An increasing number of studies showed that extracts of PSA possessed a large cohort of pharmacological functions such as anti-inflammation, anti-oxidation, anti-tumour and bacteriocidal functions [25–28]. However, the mechanisms behind the anti-inflammation effects are largely unknown owing to the absence of an effective tracking approach.

Tannins are water-soluble polyphenols, which present in many Chinese herbs and plant foods, which possess many bioactivities, such as antimicrobial, anticarcinogenic, antimutagenic, etc. In our previous experiments, we found that tannins from *Radix Paeoniae* Rubra bound greatly to lipid A and were anti-LPS *in vivo* and *in vitro*. However, this was restricted in clinical applications owing to a property of protein precipitation by intravenous administration. Herein, we first removed tannins of 12 Chinese herbs by adding excessive gelatine, and then co-incubated with the indicated LPS concentration. Finally, we tested the lipid A-binding abilities of each aqueous extraction. Our results demonstrated that the non-tannic aqueous extract from PSA bound greatly to lipid A after consumption with LPS. These findings suggest that PSA may possess abundant anti-LPS components among 12 Chinese herbs.

In order to investigate further the anti-LPS characteristics of PSA, we isolated the non-tannic aqueous extraction by affinity biosensor, polyamide chromatography and IEC–HPLC. Finally, we got a high-binding lipid A fraction from PSA (namely PSA-I-3) and tested the anti-sepsis activities *in vivo* and *in vitro*. Our results showed that PSA-I-3 could increase the survival of mice by lethal-dose LPS and heat-killed *E. coli in vivo* and inhibit significantly the TNFα release from LPS-stimulated RAW264.7 cells *in vitro*. However, PSA-I-3 could not arrest the lysis of *Limulus* amoebocytes by LPS. Instead, it enhanced LAL in spite of binding highly to lipid A The underlying mechanism is unclear, but we think that it is perhaps related to the purity of PSA-3. In conclusion, our results showed that PSA-3 possessed a potential anti-sepsis ability, and it is necessary to further investigate its bioactivities in the future.
